# In Vitro Susceptibility and Florfenicol Resistance in *Citrobacter* Isolates and Whole-Genome Analysis of Multidrug-Resistant *Citrobacter freundii*

**DOI:** 10.1155/2019/7191935

**Published:** 2019-11-19

**Authors:** Wangxiao Zhou, Qianqian Chen, Changrui Qian, Kai Shen, Xinyi Zhu, Danying Zhou, Wei Lu, Zhewei Sun, Hongmao Liu, Kewei Li, Teng Xu, Qiyu Bao, Junwan Lu

**Affiliations:** ^1^School of Laboratory Medicine and Life Science/Institute of Biomedical Informatics, Wenzhou Medical University, Wenzhou 325035, China; ^2^Tongji University School of Medicine, Shanghai 200092, China; ^3^Institute of Translational Medicine, Baotou Central Hospital, Baotou 014040, China

## Abstract

The genus *Citrobacter* is an opportunistic pathogen causing infections in animals, and the published data for its resistance to florfenicol are scarce. In this study, we investigated the antimicrobial susceptibility and molecular characteristics of florfenicol resistance genes among *Citrobacter* isolates from animal and relevant environmental samples and conducted a comparative analysis of a multidrug-resistant *Citrobacter freundii* strain isolated from a rabbit. Among 20 *Citrobacter* strains isolated from animal samples, resistance was most commonly observed to ampicillin (100%), tetracycline (75%), streptomycin (65%), florfenicol (60%), chloramphenicol (60%), and aztreonam (50%), while all the strains found in environmental samples were resistant to few antibiotics. The florfenicol resistance gene *floR* was detected in 12 isolates (48%, 12/25) from animal samples, and all of the *floR*-positive isolates were resistant to florfenicol with minimum inhibitory concentration (MIC) values ≥256 *μ*g/mL. Sequencing and comparative analysis of the plasmids from a multidrug-resistant *C. freundii* isolate named R47 showed that the *floR*-containing region in the plasmid pR47-54 was a truncated transposon-like structure and could be found on both plasmids and chromosomes of bacteria of either animal or human origin. Furthermore, a range of antimicrobial and metal resistance genes associated with mobile genetic elements could be identified in pR47-54 and the other plasmid pR47-309 of *C. freundii* R47. These results provide in-depth views into the phenotypic and molecular characteristics of *Citrobacter* isolates recovered from animal and relevant environmental samples, as well as highlight the role horizontal gene transfer plays in the dissemination of plasmid-encoded resistance genes.

## 1. Introduction

The genus *Citrobacter* is a Gram-negative, nonsporulating, and facultative anaerobic bacillus of the family *Enterobacteriaceae* [1], which at present is divided into 15 genetically distinct genomospecies: 11 previously recognized species, *C. freundii*, *C. koseri*, *C. amalonaticus*, *C. farmeri*, *C. youngae*, *C. braakii*, *C. werkmanii*, *C. sedlakii*, *C. rodentium*, *C. gillenii*, and *C. murliniae*; and four recently described species, *C. pasteurii*, *C. europaeus*, *C. bitternis*, and *C. portucalensis* [2–7], commonly found in surface waters, sewage, soil, and intestinal microflora of animals and humans. *Citrobacter* species are opportunistic pathogens particularly involving infections of the urinary and respiratory tracts in humans [8, 9]. In animals, *Citrobacter* strains are closely associated with disorders in fish, which were first described as newly emerged fish pathogens in sunfish (*Mola mola*) by Sato et al. [10]. To date, *C. freundii* has been isolated from different fish species, such as carp, Atlantic salmon, and tilapia [11–13], while *C. braakii* has been found in the gut of channel catfish and rainbow trout [14, 15]. Additionally, some of the *Citrobacter* strains are well documented to cause respiratory tract and wound infections in horses, respiratory tract infections in dogs, and pleuritis and urinary tract infections in cats [16].

With the widespread introduction of florfenicol into veterinary use for animal diseases, the resistance levels to florfenicol have increased rapidly in bacterial isolates [17, 18]. To date, 11 florfenicol resistance genes (including variants) have been identified, i.e., *floR* [19], *floRv* [20], *flost* [21], *fexA* [22], *fexB* [23], *pexA* [24], *optrA* [25], *cfr* [26], *cfr*(*B*) [27], *cfr*(*C*) [28], and *estDL136* [29]. Similar to other antimicrobial resistance (AMR) genes, the *floR* gene has been identified on numerous bacterial plasmids and chromosomes with various MGEs. For example, the complete sequence of *floR* flanked by two integrons was present in the chromosomes of several *Salmonella* serovars [19, 30]; the *floR*-containing region in the plasmid pMBSF1 of *E. coli* was composed of three parts homologous to Tn*539*3, the *floR* plasmid from *E. coli* 10660 and Tn*1721* [31], and the *floR*-flanking regions in some bacteria were associated with previously described IS*CR2* transposable elements [32].

The IncR incompatibility replicon was initially reported in a quinolone resistance plasmid, pK245, from a clinical *K. pneumoniae* isolate in 2009 [33], and IncR plasmids were mainly isolated from clinical *Enterobacteriaceae* strains carrying various resistance genes [34, 35]. In addition, IncR replicons are increasingly detected in multireplicon plasmids [36]. IncHI2 is one subgroup of the HI incompatibility group (IncHI) typically found on high molecular weight plasmids (>250 kb), which have been frequently found in *Enterobacteriaceae* isolates of human and animal origin, including *C. freundii* [37, 38]. These plasmids usually encode a range of AMR genes responsible for *β*-lactam, aminoglycoside, quinolone, and other antibiotic resistance, with many metal resistance genes located on them showing resistance to mercury, tellurite, copper, arsenic, and so on [39].

To date, there have been a number of studies unraveling the drug resistance and distribution of resistance genes among *Citrobacter* isolates in humans [40–42]; nevertheless, few studies have focused on *Citrobacter* strains isolated from animals and the environment. In this work, we sought to evaluate the drug resistance profiles and prevalence of florfenicol resistance genes among 25 *Citrobacter* isolates collected from animal and environmental samples. We also reported for the first time the complete genome of *C. freundii* from a food animal, i.e., a rabbit-derived *C. freundii* strain, R47. A comparative genomic analysis of antibiotic resistance plasmids from the strain was performed to explore the molecular mechanism of resistance dissemination.

## 2. Materials and Methods

### 2.1. Sample Collection and Bacterial Identification

Samples were collected from animals and the environment from three farms and their surroundings in Wenzhou, China, between 2016 and 2017. The former included the anal feces of chickens, ducks, geese, cattle, and rabbits as well as the intestinal tracts of fish, and the latter included soil, pond water, and sewage. Then, all of them were streaked onto LB agar plates. Bacterial species were identified using the Vitek-60 microorganism autoanalysis system (bioMerieux Corporate, Craponne, France) and 16S rDNA sequencing methods. The bacteria and plasmids used in this work are listed in Table 1.

### 2.2. Antimicrobial Susceptibility Testing

The MICs of all tested antibiotics against the bacteria were determined using the agar dilution method following the Clinical and Laboratory Standards Institute (CLSI) guidelines. The breakpoints for each antimicrobial were used according to the CLSI breakpoint criteria (CLSI, 2017) and the guidelines of the European Committee on Antimicrobial Susceptibility Testing (EUCAST, 2017). The resistance breakpoint for florfenicol (≥32 *μ*g/mL) was determined according to a previous publication for *E. coli* [43], and streptomycin (≥32 *μ*g/mL) and azithromycin (≥32 *μ*g/mL) were defined by US Food and Drug Administration (FDA) or the National Antimicrobial Resistance Monitoring System for *Salmonella* and *E. coli*. The commercially available *E. coli* strain ATCC 25922 was used as a quality control strain.

### 2.3. Detection of Florfenicol Resistance Genes

Genomic DNA was extracted from each of the *Citrobacter* strains using the AxyPrep Bacterial Genomic DNA Miniprep kits (Axygen Scientific, Union City, CA, USA) and was used as template DNA to screen for the known florfenicol resistance genes through PCR amplification. The screening primers were designed as described previously, including *floR* [44], *fexA* [45], *fexB* [23], *pexA* [46], *optrA* [25], *cfr* [47], and *estDL136* [48] primers (Table 2). Positive amplification products were purified using a MinElute PCR Purification kit (QIAGEN China, Shanghai, China) and verified by sequencing (Shanghai Sunny Biotechnology Co. Ltd., Shanghai, China). The sequencing results were analyzed and compared using the Basic Local Alignment Search Tool (BLAST) (https://blast.ncbi.nlm.nih.gov/Blast.cgi).

### 2.4. Sequencing, Assembly, and Bioinformatics Analysis

The whole genomic DNA of *C. freundii* R47 was extracted using the AxyPrep Bacterial Genomic DNA Miniprep kit (Axygen Scientific, Union City, CA, USA) and sequenced with a PacBio RS II instrument (Pacific Biosciences) and the HiSeq 2500 platform (Illumina Inc., San Diego, CA). The long PacBio reads of approximately 10-20 kb in length were initially assembled using Canu v1.6 [49], and then the processed Illumina reads were mapped onto the primary assembly to control assembly quality and to correct possible misidentified bases using bwa0.7.13, samtools1.3, and GenomeAnalysisTK2.3.9 [50, 51]. A custom-derived script written in Python (https://www.python.org/) was used to obtain the consensus sequence. Potential open reading frames (ORFs) were predicted using Glimmer3.02 with default parameters [52] and annotated against the nonredundant protein database using the BLASTX program with an *e*-value threshold of 1*e*-5. Annotation of insertion sequences and antibiotic resistance genes was performed using ISfinder and the Comprehensive Antibiotic Resistance Database (CARD) [53, 54]. Typing of *C. freundii* R47 was performed using multilocus sequence typing (MLST) with a database for *C. freundii* (https://pubmlst.org/cfreundii/). Plasmid incompatibility groups were predicted by PlasmidFinder (https://cge.cbs.dtu.dk//services/PlasmidFinder). Comparisons of the nucleotide sequences were carried out using BLASTN. Orthologous groups of genes from plasmids were identified using BLASTP and InParanoid [55]. Gview was used to construct basic genomic features that were then employed in comparative genomics analysis [56]. Additional bioinformatics tools were written using Python (https://www.python.org/) and Biopython [57].

### 2.5. Cloning Experiments

The PCR products of the AMR genes with their upstream promotor regions from *C. freundii* R47 were digested with corresponding restriction endonucleases and then ligated into suitable vectors (pUCP20 or pUCP24) (Table 3) with a T4 DNA ligase cloning kit (Takara Bio Inc., Dalian, China). The recombinant plasmids were transformed into competent *E. coli* DH5*α* cells by the calcium chloride method, and the bacterial colonies were cultured on LB agar plates supplemented with either gentamicin (20 *μ*g/mL) for pUCP24 recombinant plasmids or ampicillin (100 *μ*g/mL) for pUCP20 recombinant plasmids. The recombinant plasmids were extracted and digested with corresponding restriction enzymes to confirm the size of the inserted fragments. Each cloned fragment in the recombinant plasmids was further verified by sequencing (Shanghai Sunny Biotechnology Co. Ltd., Shanghai, China).

### 2.6. Conjugation Experiments

Conjugation experiments were performed by the filter-mating method with rifampin-resistant *E. coli* C600 (EC600) used as a recipient strain, which is aimed at detecting the conjugation potential of the plasmids of the donor *C. freundii* R47. The transconjugants were selected on two kinds of Mueller-Hinton agar plates: one containing 2,048 *μ*g/mL of rifampin and 32 *μ*g/mL of florfenicol and the other containing 2,048 *μ*g/mL of rifampin and 32 *μ*g/mL of ampicillin. The plasmids were extracted from the transconjugants and further confirmed by PCR amplification and sequencing of resistance genes on them (i.e., the presence of *floR* on pR47-54 as well as *bla*_TEM-1b_, *bla*_SHV-12_, and *bla*_DHA-1_ on pR47-309).

### 2.7. Nucleotide Sequence Accession Number

The complete nucleotide sequences of the chromosome and two plasmids of R47 in this work have been submitted to DDBJ/EMBL/GenBank, and accession numbers of the chromosome, pR47-54, and pR47-309 are CP040698, CP040697, and CP040696, respectively.

## 3. Results

### 3.1. Identification and MIC Detection of *Citrobacter* Strains

Based on biological characteristics and 16S ribosomal RNA gene homology analysis, only 25 isolates were characterized as the genus *Citrobacter* from 405 nonduplicated bacterial strains ([Supplementary-material supplementary-material-1]). Among them, 64% (16/25) were isolated from fish, 16% (4/25) from rabbits, 12% (3/25) from soil, and 8% (2/25) from sewage. These isolates consisted of 4 species, including *C. freundii* (72%, 18/25), *C. murliniae* (16%, 4/25), *C. farmeri* (8%, 2/25), and *C. gillenii* (4%, 1/25).

The MIC levels of 19 antimicrobial agents against the 25 *Citrobacter* strains are shown in Table 4. Of the 20 strains collected from the animal samples (rabbits and fish), widespread resistance was found to ampicillin (penicillin, 100%, 20/20), tetracycline (tetracycline, 75%, 15/20), streptomycin (aminoglycoside, 65%, 13/20), florfenicol (amphenicol, 60%, 12/20), chloramphenicol (amphenicol, 60%, 12/20), and aztreonam (monobactam, 50%, 10/20). In contrast, among the 5 strains of environmental origin (soil and sewage), almost no strains were resistant to any other tested antibiotic except two strains from soil (S16 was resistant to only streptomycin and aztreonam, and S30 was resistant to only tetracycline). Notably, no strains were resistant to levofloxacin, polymyxin B, fosfomycin, imipenem, or meropenem.

### 3.2. Detection of Florfenicol Resistance Genes in *Citrobacter* Strains

Among the 25 *Citrobacter* strains, 48% (12/25) were positive for the *floR* gene, but no other florfenicol resistance gene was identified. On the basis of the MIC values for the wild-type strains, all the *floR*-positive strains showed high MIC values for florfenicol (≥256 *μ*g/mL) and they were all isolated from animals (rabbits: R44, R47, R49, and R51; fish: F18, F44, HXF2, HXF4, HXF6, HXF7, HXF8, and HXF10). However, all the *floR*-negative strains remained susceptible to florfenicol (MIC values ≤ 8 *μ*g/mL), including all 5 strains of environmental origin (soil and sewage). Considering that the *floR*-positive isolates exhibited multidrug resistance phenotypes (Table 4) and that information on the genome sequences of *C. freundii* isolated from food animals was not available, one of the 4 *floR*-positive *C. freundii* isolates collected from rabbits, namely, R47, was selected for a further study.

### 3.3. General Features of the *C. freundii* R47 Genome

**Figure 1:**
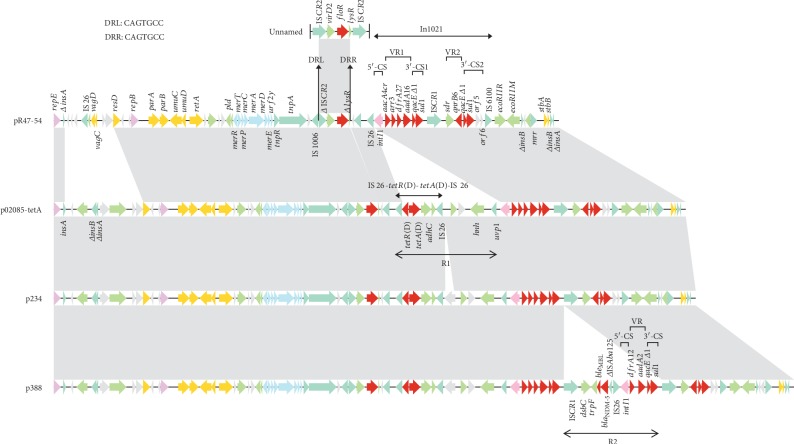
Genomic structure of the plasmid pR47-54 and comparison analysis with other sequences. The GenBank accession numbers are MH477637.1 for p02085-tetA, CP021163.1 for p234, CP021168.1 for p388, and MK191835.1 for the unnamed plasmid harboring the complete *floR*-encoding region. Genes are denoted by arrows and colored based on their assigned gene functions. DRL and DRR represent the left and right direct repeats, respectively. Shading denotes regions of homology (>95% nucleotide identity).

**Figure 2:**
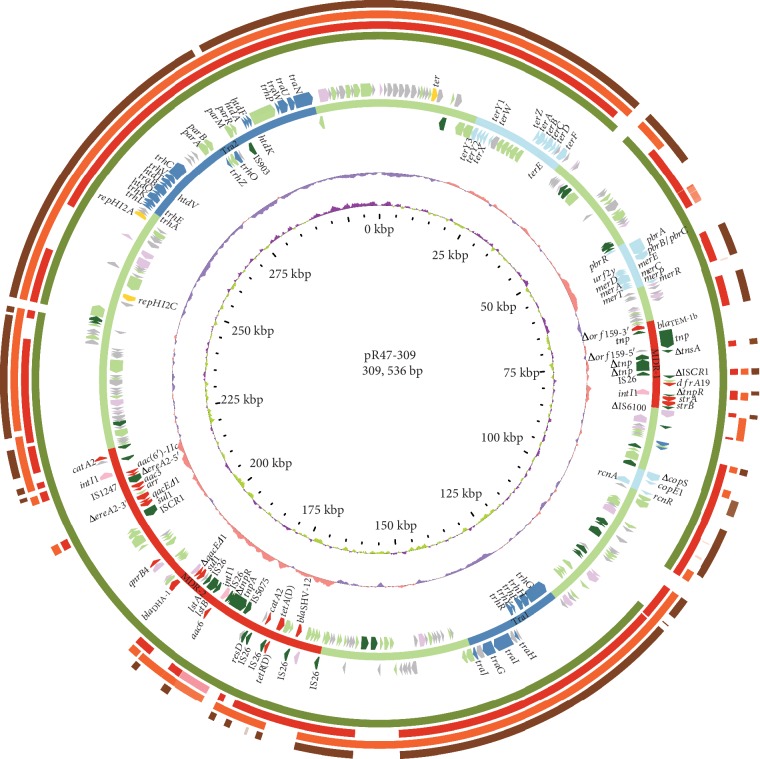
The circular map of pR47-309 and comparative genomics analysis with other IncHI2 plasmids. Circles 1-4 (from outside to inside) are homologous regions of R478 (BX664015.1), p112298-catA (KY270851.1), pT5282-mphA (KY270852.1), and p505108-MDR (KY978628.1) compared to those of pR47-309, while the regions without similar hits between them were left blank. Circle 5 and Circle 7 display genes encoded in the forward strand and reverse strand, respectively. Circles 8 and 9 represent the GC content and GC skew maps of pR47-309, respectively. Circle 10 shows the scale in kb. Circle 6 shows the different functional regions with different colors; conjugative transfer and MDR regions are marked in steel blue and red, respectively, which are further denoted within the related bars; heavy metal resistance regions are marked in light blue, and other functional regions are in light green.

The genome of *C. freundii* R47 consists of a circular chromosome and two antibiotic resistance plasmids designated pR47-54 and pR47-309 (Table 5 and Figures 1 and 2). The chromosome is approximately 4.95 Mb in length and contains 4,548 ORFs with an average GC content of 51.70%. MLST allowed assignment of the R47 to a new *C. freundii* sequence type: ST263. The plasmids pR47-54 and pR47-309 have circularly closed DNA sequences of 53,964 bp and 309,536 bp in length with average GC contents of 54.25% and 47.71%, respectively. The plasmid pR47-54 harbors 70 ORFs and could be assigned to the incompatibility group IncFIA/IncR, while pR47-309 carries 369 ORFs and belongs to the incompatibility group IncHI2. Based on functional annotation of the ORFs, a total of 31 antibiotic resistance genes were identified in the genome sequences of the chromosome (1/31), pR47-54 (10/31), and pR47-309 (20/31) (Table 6), which were involved in resistance to 9 classes of antibiotics (*β*-lactams, amphenicols, aminoglycosides, quinolones, sulfonamides, trimethoprims, tetracyclines, rifampicins, and macrolides). Notably, of these AMR genes, *bla*_CMY-97_, *aadA16*, and *dfrA19* were first reported in *Citrobacter*, as well as *catA2*, *bla*_DHA-1_, *aac6*, and *qnrB4* were reported in animal-derived *C. freundii* for the first time. The antibiotic resistance genes in pR47-309 were located in two separated multidrug-resistant (MDR) regions, MDR-1 and MDR-2 (Figure 2). Moreover, all of the AMR genes in two plasmids were associated with various MGEs. In addition, five complete or incomplete clusters of heavy metal resistance genes (the resistance was related to mercury, tellurite, lead, copper, and nickel/cobalt) were identified in pR47-309, while a complete mercury resistance operon (*merEDACPTR*) was found in pR47-54 (Figures 1 and 2 and Table 6). Conjugation experiments were unsuccessful for both plasmids.

### 3.4. Functional Detection of the Resistance Genes in *C. freundii* R47

The MIC levels of nineteen antimicrobial agents against the recombinant strains expressing the cloned resistance genes are shown in Table 7. In addition to *qnrB4* and *qnrB6*, other resistance genes (*floR*, *catA2*, *bla*_CMY-97_, *bla*_DHA-1_, *bla*_SHV-12_, *bla*_TEM-1b_, *strA*, *strB*, *aac6*, *aac3*, *aacA4cr*, and *aac(6*′*)-IIc*) were all functional. The recombinant strains harboring cloned *floR* exhibited an at least 8-fold increase in MIC levels to both florfenicol and chloramphenicol, while the recombinant strains with cloned *catA2* exhibited a 128-fold increase to only chloramphenicol, compared with those of the control strains (*E. coli* DH5*α* harboring the vector pUCP24 or pUCP20). The 4 recombinant strains expressing the cloned *bla* genes (pUCP24*-bla*_CMY-97_*/*DH5*α*, pUCP24*-bla*_DHA-1_*/*DH5*α*, pUCP24*-bla*_SHV-12_*/*DH5*α*, and pUCP24*-bla*_TEM-1b_*/*DH5*α*) showed a more than 32-fold increase in MIC levels to benzylpenicillin and ticarcillin, with at least 256-, 16-, 4-, and 8-fold increases to ampicillin, piperacillin, piperacillin/tazobactam, and a first-generation cephalosporin (cefazolin), respectively. Moreover, apart from the recombinant strains with cloned *bla*_TEM-1b_, the recombinant strains with the other three cloned *bla* genes also exhibited respective >64- and >4-fold increases to a third-generation cephalosporin (ceftazidime) and aztreonam, respectively, and only the recombinants expressing the *bla*_SHV-12_ gene show resistance to a fourth-generation cephalosporin (cefepime). The results of the functional detection of the cloned resistance genes against aminoglycoside antibiotics demonstrated that the MIC values of streptomycin were 64- and 4-fold higher in recombinants carrying *strA* and *strB*, respectively, and that the recombinants harboring one of the remaining four aminoglycoside resistance genes (*aac6*, *aac3*, *aacA4cr*, and *aac(6*′*)-IIc*) displayed resistance to some of the other 4 aminoglycoside antibiotics (kanamycin, gentamicin, amikacin, and tobramycin).

### 3.5. Comparative Genomics Analysis of the *floR*-Carrying Plasmid pR47-54

A search of the nr/nt database revealed that pR47-54 shared the highest nucleotide sequence similarity (93% coverage and 99% identity) with three IncFIA/IncR plasmids, namely, p388 from *E. cloacae* strain 388 (CP021168.1, 79 kb) isolated from the USA, p234 from *E. cloacae* strain 234 (CP021163.1, 68 kb) isolated from the USA, and p02085-tetA from *C. freundii* strain 1509-02085 (MH477637.1, 67 kb) isolated from northern China (Figure 1). Comparative genomics analysis showed that pR47-54 shared the same number of homologous genes (62/70, the identity of amino acid sequences was between 93.82 and 100%) with the three plasmids p388, p234, and p02085-tetA, including all the AMR genes and a complete mercury resistance operon (*merEDACPTR*). As shown in Figure 1, pR47-54 displayed highly global genomic synteny and shared a conserved backbone sequence of typical IncR plasmids [58] with the three aforementioned plasmids, which included *repB* for replication initiation, *parAB* for partition, *umuCD* likely for SOS mutagenesis, *resD* for maintenance, and *retA* for reverse transcription; however, the *vagCD* toxin-antitoxin system was present in only pR47-54. Additionally, in comparison with the sequence of pR47-54, there were some insertions in the three plasmids, e.g., an insertion of R1 downstream of *intI1* was found in the three plasmids, which was comprised of a tetracycline resistance unit (IS*26*-*tetR*(D)-*tetA*(D)-IS*26*) (i.e., Tn*tet*(D) [59]) and other genes with known or unknown functions, and the insertion sequence R2 upstream of IS*CR1* was identified in p388, which consisted of an IS*CR1*-mediated unit (including two AMR genes, namely, *ble*_MBL_ and *bla*_NDM-16_), a truncated IS*Aba125*, an IS*26*, and a class 1 integron (sequentially organized as a 5′-conserved segment (5′-CS: *intI1*) variable region (VR: *dfrA12* and *aadA2*) and 3′-conserved segment (3′-CS: *qacEΔ1* and *sul1*)) (Figure 1).

Further analysis showed that the *floR*-related region (*Δ*IS*CR2*-*virD2*-*floR*-*ΔlysR*) in pR47-54 was also located in transposon-like fragments, which were bracketed by a pair of complete or truncated IS*CR2* elements (IS*CR2* (*Δ*IS*CR2*)-*virD2*-*floR*-*lysR*-IS*CR2* (*Δ*IS*CR2*)) (Figure 1), of a large number of plasmids and chromosomes of the various host strains from different origins, such as an unnamed plasmid from *S. enterica* subsp. *enterica* serovar Braenderup strain 76 (MK191835.1), the plasmid pHN6DS2 from *E. coli* strain GZ6DS2 (MH459020.1), the chromosome of *P. mirabilis* strain PmSC1111 (CP034090.1) of animal origin, as well as the plasmid pK1HV from *K. pneumoniae* strain K1HV (HF545434.1), the plasmid pSLK172-1 from *E. coli* strain SLK172 (CP017632.1), and the chromosome of *A. baumannii* strain MRSN15313 (CP033869.1) of human origin. In addition, a pair of 7 bp perfect direct repeats (DRs; CAGTGCC) immediately flanked this *floR*-related region in pR47-54.

### 3.6. Comparative Genomics Analysis of the IncHI2 Plasmid pR47-309 Genome

**Figure 3:**
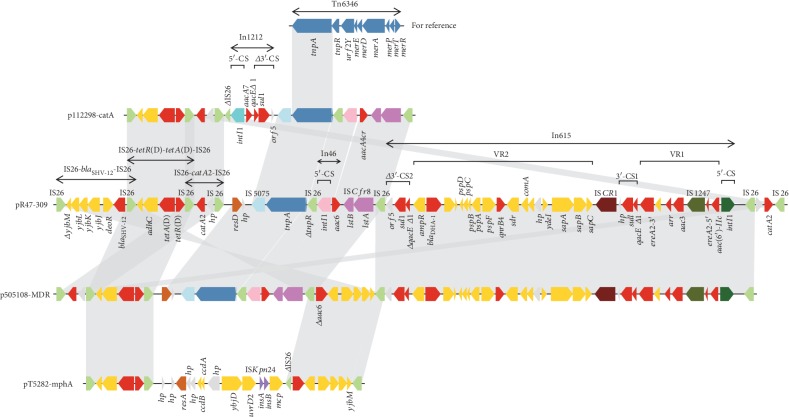
Comparison of the MDR-2 region from pR47-309 with other related regions. Genes are denoted by arrows and colored according to gene function classification. Shading indicates the regions of homology (>95% nucleotide identity). The accession numbers are KY270851.1 for p112298-catA, KY978628.1 for p505108-MDR, and KY270852.1 for pT5282-mphA.

To further characterize the similarities and differences among several genomes of IncHI2 plasmids, we performed a comparative genomic analysis of pR47-309 with four other representative IncHI2 plasmids isolated from clinical strains. The results showed that pR47-309 shared a nucleotide sequence identity of 99% with all of them, including p505108-MDR of *C. sakazakii* (KY978628.1, 312 kb) at 93% coverage, pT5282-mphA of *E. cloacae* (KY270852.1, 282 kb) at 73% coverage, p112298-catA of *C. freundii* (KY270851.1, 263 kb) at 72% coverage, and R478 of *S. marcescens* (BX664015.1, 274 kb) at 67% coverage (Figure 2). Further analysis showed that pR47-309 shared the maximum homologous genes (>90% similarity of amino acid sequences) with the largest plasmid, p505108-MDR (91.06%, 336/369), whereas only 65.85% (243/369), 64.77% (239/369), and 60.43% (223/369) of the genes of it showed high similarity (>90%) with those of the remaining three plasmids, pT5282-mphA, p112298-catA, and R478, respectively. Furthermore, these five plasmids shared the core backbone determinants of typical IncHI2 plasmids [60], including the replicons (*repHI2A* and *repHI2C*), the Tra1 and Tra2 conjugative transfer regions, several plasmid partition genes (*parABMR*) within Tra2, and the tellurite resistance region (*terY3Y2XY1WZABCDEF*). In addition, each of the plasmids possessed their own VRs, mainly including the heavy metal resistance gene clusters and accessory modules containing AMR genes. In terms of the former, for instance, a complete mercury resistance region (*merEDACPTR*) was present in all these plasmids except p112298-catA, while an incomplete copper operon consisting of *ΔpcoS* and *pcoE1* as well as a nickel/cobalt efflux system (*rcnA*/*rcnR*) was identified in pR47-309, p505108-MDR, and pT5282-mphA. Of note, the partial lead resistance *pbr* gene cluster (*pbrB*/*C*, *pbrA*, and *pbrR*) was harbored by only pR47-309 and p505108-MDR. Regarding the latter, the entire MDR-1 of pR47-309 could be identified in p505108-MDR [61]; however, the MDR-2 of pR47-309 was a highly complex mosaic structure, harboring 6 resistance-related units (IS*26*-*bla*_SHV-12_-IS*26*, IS*26*-*tetR*(D)-*tetA*(D)-IS*26*, IS*26*-*catA2*-IS*26*, In46, In615, and IS*26*-*catA2*-IS*26*), all of which could also be discovered in p505108-MDR but with different arrangements of the units (a sequence of 16 bp was deleted in the middle of In46). Simultaneously, partial units of MDR-2 could be found in p112298-catA (*aac6* was replaced by *aacA4cr* compared in pR47-309) and pT5282-mphA (Figure 3).

## 4. Discussion

In this work, based on both biochemical and molecular methods (16S rRNA sequencing), a total of 25 isolates were identified as *Citrobacter* species among 405 bacterial strains. It is interesting that most of them (64%, 16/25) were isolated from fish, indicating that the prevalence of *Citrobacter* strains is increasing among aquaculture species. Although *C. freundii* accounts for majority of strains (85%, 17/20) collected from farm animals, one *C. gillenii* and two *C. murliniae* isolates were also found from fish, suggesting a variety of members of the *Citrobacter* genus distributed in fish. Notably, according to the MIC results, all 25 *Citrobacter* strains were sensitive to levofloxacin, polymyxin B, fosfomycin, imipenem, and meropenem. However, a few carbapenem resistance phenotypes have been detected in some *Citrobacter* strains isolated from clinical or clinic-associated samples such as hospital sewage, including KPC-2, NDM-1, and OXA-48 [62, 63]. No information is available about the presence of any carbapenemase-producing *Citrobacter* strains collected at the farms or their surroundings, which may be a result of the prohibition of the use of carbapenems in food animals worldwide. On the other hand, high resistance rates for ampicillin, tetracycline, and streptomycin among the strains of animal origin in this work may mainly be induced by overusing these antibiotics in animal farming. The resistance rate for florfenicol of *Citrobacter* strains isolated from fish in our study (50%, 8/16) was significantly higher than that from fish collected from the Aegean, Central Anatolia, and Mediterranean Sea regions (28.57%, 2/7) [64], reflecting a rapid extension of off-label use of florfenicol for aquaculture in China.

Of note, our MIC results for *Citrobacter* strains isolated from animal samples greatly differed from those for 385 strains of clinical origin reported in a previous analysis from the University Hospital of Heraklion, Crete, Greece, during a six-year period (2010-2015) [40]. Specifically, higher resistance rates were detected among the former for most common antimicrobial agents, while identical or slightly lower susceptibilities for ampicillin (100%), carbapenems (98.7%-99.0%), levofloxacin (93.2%), and fosfomycin (99.2%) were observed among the latter. An at least 12-fold increase in the prevalence of resistance to chloramphenicol, tetracycline, gentamicin, and tigecycline was observed in our study. A similar report demonstrated that among sixty-four clinical *Citrobacter* isolates tested for susceptibility from humans of China, a decrease to the resistance rates for chloramphenicol, gentamicin, and tetracycline by 25-, 17-, and 9-fold, respectively, was observed relative to those of animal-derived *Citrobacter* strains in our work, though higher resistance rates for ceftazidime (29.3%) and levofloxacin (2.4%) were seen in the former [41]. These findings suggested that the emergence and spread of drug resistance among *Citrobacter* strains from food animal sources was probably more serious than that of humans; therefore, reasonable use of antibiotics in farm animals is a task that brooks no delay.

The positive rate for *floR* among florfenicol-resistant *Citrobacter* strains of our study (100%, 12/12) was observably higher than that of 119 florfenicol-resistant Gram-negative bacilli from seven freshwater Chilean salmon farms (21.8%, 26/119) [65], and each of the *floR*-positive strains was isolated from animal samples. Nonetheless, all the florfenicol-susceptible strains in our study lacked *floR*, including all the strains of environmental origin. This finding suggested that the resistance to florfenicol of these *Citrobacter* strains was closely related to the presence of *floR*.

In the plasmid pR47-54, we identified 7 bp DRs flanking the *floR*-encoding region (*Δ*IS*CR2*-*virD2*-*floR*-Δ*lysR*), suggesting that this region was formed by a transposition process and that the deletion of the border sequences of this region occurred posttransposition. Furthermore, sequences containing this region in various plasmids and chromosomes of bacteria of animal or human origin were identified with two complete or truncated IS*CR2* flanking them (IS*CR2* (ΔIS*CR2*)-*virD2*-*floR*-*lysR*-IS*CR2* (ΔIS*CR2*)). This finding demonstrated that the *floR*-encoding region in pR47-54 might be formed by the IS*CR2*-mediated transposon-like structure and that IS*CR2* enabled the wide dissemination of *floR*-encoding sequences among a variety of bacteria of animal and human origins, which was in line with a previous study about the association of IS*CR2* with *floR* [32].

Comparative genomics analysis of pR47-54 with three other IncFIA/IncR plasmids (p388, p234, and p02085-tetA) revealed that despite there were some insertions in the latter plasmids compared with the sequence of pR47-54, the backbone components of the four plasmids remained unchanged, except for *vagCD* encoding a toxin-antitoxin system in only pR47-54. This result suggested that these three plasmids probably evolved from pR47-54-like plasmids. Furthermore, the host bacteria of these plasmids originated from the USA, northern China, and southern China, indicating a global distribution of the IncFIA/IncR plasmids. Although pR47-54 is nonconjugative due to lack of a conjugative transfer system, all of its resistance genes were flanked by MGEs, implying that they could be spread to other transmissible plasmids by HGT. The common backbone of pR47-309 with four other representative IncHI2 plasmids of clinical isolates (p505108-MDR, pT5282-mphA, p112298-catA, and R478) indicated that transmission of IncHI2 plasmids occurs between strains of animal and human origins. Moreover, insertions, deletions, or rearrangements of resistance genes with related MGEs may explain the diversity of genomes among the five IncHI2 plasmids. There were eight copies of intact IS*26* located in MDR-2 of pR47-309, and the IS26-flanking units lacked DRs at both ends, which could mediate future events for assembly of resistance genes through homologous recombination to build a MDR region with a complex chimera structure [66] (Figure 3).

Extended-spectrum *β*-lactamase (ESBL) and AmpC genes in *Citrobacter* species have been widely reported [63, 67], including *bla*_SHV_, *bla*_TEM_, *bla*_CTX_, and *bla*_PER_ for the former and *bla*_CMY_, *ampR*, and *bla*_DHA_ for the latter. Interestingly, this study is the first description of *bla*_CMY-97_ and *bla*_DHA-1_ in *Citrobacter* and animal-borne *C. freundii*, respectively. Moreover, *bla*_CMY_-type, *bla*_TEM_-type, *bla*_SHV_-type, and *bla*_DHA_-type genes, in particular the latter three, were found to be encoded in one *Citrobacter* isolate and one *Citrobacter* plasmid for the first time, respectively. Quinolone resistance *qnr* genes and aminoglycoside resistance genes have been identified in *Citrobacter* species as well [68, 69], and the *qnrB* genes constitute the most predominant and variable group within the *qnr* family [70]. To the best of our knowledge, this work represents the first report of *aadA16* in *Citrobacter*, as well as *qnrB4* and *aac6* in animal-associated *C. freundii*. To date, only one study has explicitly described the occurrence of *catA2* in *Citrobacter* species, which is located on the aforementioned plasmid p112298-catA from a clinical strain [71]. Therefore, we reported, for the first time, the detection of *catA2* in a *Citrobacter* isolate of animal origin. In addition to encoding AMR genes, the two plasmids pR47-54 and pR47-309 also harbored clusters of genes conferring resistance to heavy metals (mercury, tellurite, copper, nickel/cobalt, and lead). In fact, metal contamination may lead to the proliferation of antibiotic resistance [72]. These findings revealed the important role pR47-309 and pR47-54 may play in the dissemination of resistance determinants under the selective pressure of antibiotics and metals.

## 5. Conclusion

Our study demonstrated that the antimicrobial susceptibility profiles of *Citrobacter* strains collected from animals were significantly different from those of strains of either environmental origin or human origin. In addition, a large proportion of the *Citrobacter* strains of animal origin were resistant to most of the tested antibiotics, most notably ampicillin, tetracycline, and streptomycin. The high positive rate for *floR* in *Citrobacter* strains isolated from animal samples (60%, 12/20) was probably due to the off-label use of florfenicol in animals at farms. The whole-genome analysis of an animal-originated *C. freundii* isolate with two plasmids (pR47-54 and pR47-309) revealed that the *floR* gene was associated with a truncated transposon-like structure on the plasmid pR47-54 that could be identified in both plasmids and chromosomes of isolates of animal and human origins, which suggested the potential dissemination of *floR* from animals to humans. Moreover, the circulation of numerous resistance genes associated with MGEs in the plasmids pR47-54 and pR47-309 may be promoted by HGT under the selective pressure of antibiotics and metals. Therefore, active surveillance and monitoring of the use of antibiotics in animals and plasmid-mediated multidrug resistance in *Citrobacter* strains of animal origin is urgently needed.

## Figures and Tables

**Table 1:** Bacteria and plasmids used in this work.

Strain or plasmid	Relevant characteristic(s)^a^	Reference or source
Strain		
25 *Citrobacter* strains ([Supplementary-material supplementary-material-1])	25 *Citrobacter* strains from 405 nonduplicated bacterial isolates	This study
DH5*α*	*Escherichia coli* DH5*α* was used as a host for the cloned resistance genes	Our laboratory collection
ATCC25922	*Escherichia coli* ATCC25922 was used as the quality control for antimicrobial susceptibility testing	Our laboratory collection
pUCP24-ORFs/DH5*α*	DH5*α* carrying the recombinant plasmids of pUCP24 cloned with resistance gene ORFs with their upstream promoter regions (*floR*, *bla*_CMY-97_, *bla*_DHA-1_, *bla*_SHV-12_, *bla*_TEM-1b_, *qnrB4*, and *qnrB6*)	This study
pUCP20-ORFs/DH5*α*	DH5*α* carrying the recombinant plasmids of pUCP20 cloned with resistance gene ORFs with their upstream promoter regions (*catA2*, *strA*, *strB*, *aac6*, *aac3*, *aacA4cr*, and *aac(6*′*)-IIc*)	This study
EC600	*Escherichia coli* C600 was used as the recipient for conjugation experiment; RIF^r^	Our laboratory collection
Plasmid		
pUCP20	Cloning vector for the PCR products of resistance gene ORFs with the promoter regions; AMP^r^	Our laboratory collection
pUCP24	Cloning vector for the PCR products of resistance gene ORFs with the promoter regions; GEN^r^	Our laboratory collection

^a^ORFs: open reading frames; r: resistance; RIF: rifampin; AMP: ampicillin; GEN: gentamicin.

**Table 2:** Primers used for the detection of florfenicol resistance genes in this work.

Target gene	Primer	Primer sequence (5′-3′)	Amplicon size (bp)	Reference
*floR*	*floR*-F	GGCTTTCGTCATTGCGTCTC	650	Zhang et al. [44]
	*floR*-R	ATCGGTAGGATGAAGGTGAGGA		
*fexA*	*fexA*-F	GTACTTGTAGGTGCAATTACGGCTGA	1,272	Kehrenberg and Schwarz [45]
	*fexA*-R	CGCATCTGAGTAGGACATAGCGTC		
*fexB*	*fexB*-F	TTCCCACTATTGGTGAAAGGAT	816	Liu et al. [23]
	*fexB*-R	GCAATTCCCTTTTATGGACGTT		
*pexA*	*pexA*-F	ACAGTGCAGGTCGAAGAACC	215	Zhao et al. [46]
	*pexA*-R	TGCATTACCAATCGACATCC		
*optrA*	*optrA*-F	AGGTGGTCAGCGAACTAA	1,395	Wang et al. [25]
	*optrA*-R	ATCAACTGTTCCCATTCA		
*Cfr*	*cfr*-F	TGAAGTATAAAGCAGGTTGGGAGTCA	746	Kehrenberg and Schwarz [47]
	*cfr*-R	ACCATATAATTGACCACAAGCAGC		
*estDL136*	*estDL136*-F	TGCCCGCACCCGATTTCT	864	Zhang et al. [48]
	*estDL136*-R	GATTGGATGCACCTCGTTCTA		

**Table 3:** Primers used for functional detection of the resistance genes in this work.

Target gene	Primer	Sequence (5′-3′)^a^	Restriction endonuclease	Vector	Annealing temperature (°C)	Amplicon size (bp)
*floR*	*floR*-F	GCTCTAGATTAGGGCGGCAGAGGGGGCTGGAAC	*Xba*I	pUCP24	64	1,477
	*floR*-R	CCAAGCTTTTAGACGACTGGCGACTTCTCGGTG	*Hin*dIII			
*catA2*	*catA2*-F	CGGAATTCAATAAAACCGGGCTTAATACAGATT	*Eco*RI	pUCP20	58	808
	*catA2*-R	GCTCTAGATTATTTCAGTATGTTATCACACATC	*Xba*I			
*bla*_CMY-97_	*bla*_CMY-97_-F	GCTCTAGATTATTATGGGTAGAAATATGCAAAT	*Xba*I	pUCP24	58	1,688
	*bla*_CMY-97_-R	CCAAGCTTTTATTGCAGTTTTTCAAGAATGCGC	*Hin*dIII			
*bla*_DHA-1_	*bla*_DHA-1_-F	GCTCTAGATAATCCACCTGTAAGTTTTTCTTTA	*Xba*I	pUCP24	58	1,267
	*bla*_DHA-1_-R	CCAAGCTTTTATTCCAGTGCACTCAAAATAGCC	*Hin*dIII			
*bla*_SHV-12_	*bla*_SHV-12_-F	GCTCTAGAATGGGTTCATGTGCAGCTCCATCAG	*Xba*I	pUCP24	58	1,023
	*bla*_SHV-12_-R	CCAAGCTTTTAGCGTTGCCAGTGCTCGATCAGC	*Hin*dIII			
*bla*_TEM-1b_	*bla*_TEM-1b_-F	GCTCTAGAAGTATTGCCCGCTCCACGGTTTATA	*Xba*I	pUCP24	58	1,113
	*bla*_TEM-1b_-R	CCAAGCTTTTACCAATGCTTAATCAGTGAGGCA	*Hin*dIII			
*strA*	*strA*-F	CGGAATTCCGGCCTGGTCCTTCAGCCACCATGC	*Eco*RI	pUCP20	58	1,590
	*strA*-R	GCTCTAGATCAACCCCAAGTCAGAGGGTCCAAT	*Xba*I			
*strB*	*strB*-F	CGGAATTCGAACGAGAGCTACCGGTGCGGCTCG	*Eco*RI	pUCP20	58	1,167
	*strB*-R	GCTCTAGACTAGTATGACGTCTGTCGCACCTGC	*Xba*I			
*aac6*	*aac6*-F	CGGAATTCAGGTTGCCGGGTGACGCACACCGTG	*Eco*RI	pUCP20	58	913
	*aac6*-R	GCTCTAGATTAGGCATCACTGCGTGTTCGCTCG	*Xba*I			
*aac3*	*aac3*-F	CGGAATTCATCGCGATCCACGCTCAAACTGAAC	*Eco*RI	pUCP20	58	1,068
	*aac3*-R	GCTCTAGATCAGGGCGAGCCAAAGTGCCGTTGA	*Xba*I			
*aacc4cr*	*aacA4cr*-F	CGGAATTCGGCGGTTTTCATGGCTTGTTATGAC	*Eco*RI	pUCP20	58	874
	*aacA4cr*-R	GCTCTAGATTAGGCATCACTGCGTGTTCGCTCG	*Xba*I			
*aac(6*′*)-IIc*	*aac(6*′*)-IIc*-F	CGGAATTCACGCACACCGTGGAAACGGATGAAG	*Eco*RI	pUCP20	58	913
	*aac(6*′*)-IIc*-R	GCTCTAGATCATGACCACTTCCCCTTGATTTTG	*Xba*I			
*qnrB4*	*qnrB4*-F	GGGGTACCCCCTACCGCTGGATCTGCGTGAATT	*Kpn*I	pUCP24	62	901
	*qnrB4*-R	CCAAGCTTTTAACCCATGACAGCGATACCAAGA	*Hin*dIII			
*qnrB6*	*qnrB6*-F	CGGAATTCGCCAGCCTTTCATGATATATCTCCC	*Eco*RI	pUCP24	58	904
	*qnrB6*-R	CGGGATCCCTAACCAATCACCGCGATGCCAAGC	*Bam*HI			

^a^The underlined sequences represent the restriction endonuclease sites and their protective bases.

**Table 4:** MIC values for 25 wild *Citrobacter* strains (*μ*g/mL).

Strains	FFC	CHL	AMP	CAZ	FEP	IPM	MEC	ATM	NAL	CIP	LVX	GEN	STR	KAN	AZM	TCY	TGC	FOS	PB
ATCC25922	4	4	4	32	<1	4	128	<1	<1	<0.5	<0.5	8	<1	4	<1	<1	<1	<1	<1
R44	256	512	>1,024	16	<1	<1	<1	32	16	<0.5	<0.5	512	256	128	<1	128	<1	8	<1
R47	256	256	>1,024	32	<1	<1	<1	32	16	<0.5	<0.5	512	256	128	<1	128	2	8	<1
R49	256	256	512	16	<1	<1	<1	64	16	<0.5	<0.5	<1	128	16	2	128	<1	8	<1
R51	256	256	>1,024	16	<1	<1	<1	64	16	<0.5	<0.5	512	128	128	<1	128	<1	8	<1
F18	256	64	128	<1	<1	<1	<1	<1	8	<0.5	<0.5	<1	512	128	<1	128	<1	16	<1
F28	8	8	64	<1	<1	<1	<1	<1	8	<0.5	<0.5	<1	8	2	<1	32	<1	16	2
F30	4	4	64	<1	<1	<1	<1	<1	>1,024	<0.5	<0.5	<1	32	<1	<1	64	<1	16	<1
F32	4	4	1,024	<1	<1	<1	<1	64	>1,024	<0.5	<0.5	<1	16	<1	<1	64	<1	16	<1
F39	8	8	128	<1	<1	<1	<1	<1	8	<0.5	<0.5	<1	16	32	<1	4	<1	4	<1
F44	256	256	>1,024	16	<1	<1	<1	64	16	<0.5	<0.5	256	128	128	<1	128	<1	8	<1
F49	8	4	64	<1	<1	<1	<1	16	8	<0.5	<0.5	<1	4	<1	<1	8	<1	8	<1
F57	8	4	64	<1	<1	<1	<1	<1	8	<0.5	<0.5	<1	8	<1	<1	4	<1	8	<1
F60	8	4	64	<1	<1	<1	<1	<1	8	<0.5	<0.5	<1	16	<1	<1	4	<1	8	<1
F61	8	4	64	<1	<1	<1	<1	<1	8	<0.5	<0.5	<1	16	<1	<1	4	<1	16	<1
HXF2	1,024	256	1,024	<1	<1	<1	<1	<1	>1,024	>8	>4	<1	>1,024	>1,024	16	256	2	8	<1
HXF4	1,024	512	1,024	<1	<1	<1	<1	<1	>1,024	>8	>4	512	512	64	64	256	2	32	<1
HXF6	>1,024	512	>1,024	32	16	<1	<1	128	>1,024	>8	>4	512	1,024	>1,024	32	512	<1	8	<1
HXF7	512	512	>1,024	32	16	<1	<1	128	>1,024	>8	2	512	512	>1,024	32	512	2	8	<1
HXF8	1,024	512	>1,024	8	<1	2	<1	64	>1,024	>8	>4	512	1,024	32	64	256	8	16	<1
HXF10	1,024	512	128	<1	<1	<1	<1	<1	>1,024	4	2	<1	128	32	>1,024	256	8	16	<1
S3	8	4	8	<1	<1	<1	<1	<1	4	<0.5	<0.5	<1	16	2	<1	4	<1	16	<1
S16	8	4	8	<1	<1	<1	<1	64	8	<0.5	<0.5	<1	32	2	<1	4	<1	32	<1
S30	8	4	8	<1	<1	<1	<1	8	8	<0.5	<0.5	<1	16	2	<1	256	<1	8	<1
A14	8	4	16	<1	<1	<1	<1	8	4	<0.5	<0.5	<1	4	<1	<1	4	<1	32	<1
A15	8	4	16	<1	<1	<1	<1	<1	8	<0.5	<0.5	<1	4	<1	<1	4	<1	32	<1

FFC: florfenicol; CHL: chloramphenicol; AMP: ampicillin; CAZ: ceftazidime; FEP: cefepime; IPM: imipenem; MEC: meropenem; ATM: aztreonam; NAL: nalidixic acid; CIP: ciprofloxacin; LVX: levofloxacin; GEN: gentamicin; STR: streptomycin; KAN: kanamycin; AZM: azithromycin; TCY: tetracycline; TGC: tigecycline; FOS: fosfomycin; PB: polymyxin B.

**Table 5:** General features of *C. freundii* R47 genome.

	Chromosome	pR47-54	pR47-309
Size (bp)	4,952,107	53,964	309,536
GC content (%)	51.70	54.25	47.71
ORFs	4,548	70	369
Known proteins	4,050 (89.1%)	57 (81.4%)	253 (68.6%)
Hypothetical proteins	498 (10.9%)	13 (18.6%)	116 (31.4%)
Protein coding (%)	88.01	80.35	83.88
Average ORF length (bp)	958	619	703
Average protein length (aa)	318	205	233
tRNAs	84	0	0
rRNA operons	(16S-23S-5S) ∗7	0	0
	16S-23S-5S-5S		

**Table 6:** Resistance genes encoded on the *C. freundii* R47 genome.

Genome	Class of resistance genes	Resistance genes
Chromosome	*β*-Lactam	*bla*_CMY-97_
pR47-54	Amphenicol	*floR*
	Quinolone	*qnrB6*
	Aminoglycoside	*aadA16* and *aacA4cr*
	Trimethoprim	*dfrA27*
	Quaternary ammonium compounds	2*qacEΔ1*
	Sulfonamide	*2sul1*
	Rifampin	*Arr-3*
	Mercury	*merE*, *merD*, *merA*, *merC*, *merP*, *merT*, and *merR*
pR47-309	*β*-Lactam	*bla*_TEM-1b_, *bla*_SHV-12_, and *bla*_DHA-1_
	Quinolone	*qnrB4*
	Tetracycline	*tetA*(D) and *tetR*(D)
	Aminoglycoside	*aac3*, *aac(6*′*)-IIc*, *aac6*, *strA*, and *strB*
	Macrolide	*ereA2*
	Sulfonamide	2*sul1*
	Quaternary ammonium compounds	*qacEΔ1* and *Δ*qacE*Δ*1
	Trimethoprim	*dfrA19*
	Rifampin	*Arr*
	Amphenicol	2*catA2*
	Tellurite	*terY3*, *terY2*, *terX*, *terY1*, *terW*, *terZ*, *terA*, *terB*, *terC*, *terD*, *terE*, and *terF*
	Mercury	*merE*, *merD*, *merA*, *merC*, *merP*, *merT*, and *merR*
	Copper	Δ*copS* and *copE1*
	Lead	*pbrR*, *pbrA*, and *pbrB/pbrC*
	Nickel/cobalt	*rcnA* and *rcnR*

**Table 7:** MIC values for transformants expressing cloned resistance genes.

Strains	FFC	CHL	PG	AMP	TIC	PRL	PTZ	CFZ	FOX	CAZ	FEP	ATM	STR	KAN	GEN	AMK	TOB	NAL	CIP
ATCC 25922	2	4	32	8	4	2	2	2	2	0.125	0.25	0.25	8	4	1	2	0.5	2	0.03
DH5*α*	4	4	16	4	2	2	2	2	2	0.125	0.25	0.25	4	2	1	1	0.25	4	0.015
pUCP20/DH5*α*	4	4	>1,024	>1,024	128	32	32	32	4	0.125	1	0.5	4	2	0.5	1	0.25	4	0.015
pUCP24/DH5*α*	4	4	32	4	1	0.5	1	4	4	0.125	0.06	0.125	2	1	128	1	4	64	0.06
pUCP24-*floR*/DH5*α*	128	32	16	4	1	2	2	1	64	0.125	0.125	0.125	8	4	64	1	0.5	4	0.06
pUCP20-*catA2*/DH5*α*	4	512	>1,024	>1,024	128	32	32	16	8	0.125	1	0.5	4	2	1	2	0.5	4	0.015
pUCP24-*bla*_CMY-97_/DH5*α*	4	4	>1,024	>1,024	128	32	8	128	<1	>8	0.5	>1	2	1	32	<0.125	1	4	<0.0075
pUCP24-*bla*_DHA-1_/DH5*α*	2	4	>1,024	1,024	128	8	16	64	16	>8	0.25	>1	4	2	32	1	0.5	4	<0.0075
pUCP24-*bla*_SHV-12_/DH5*α*	2	1	>1,024	>1,024	128	64	64	256	4	>8	>4	>1	1	0.25	32	1	0.5	2	0.015
pUCP24-*bla*_TEM-1b_/DH5*α*	2	1	>1,024	>1,024	128	64	4	32	<1	0.125	0.5	0.25	1	0.5	32	<0.125	0.5	2	<0.0075
pUCP20-*strA*/DH5*α*	2	4	>1,024	>1,024	128	32	32	16	4	0.125	0.5	0.5	256	2	0.5	1	0.25	4	<0.0075
pUCP20-*strB*/DH5*α*	2	4	>1,024	>1,024	128	32	32	16	2	0.125	0.5	0.5	16	2	1	2	0.5	4	<0.0075
pUCP20-*aac6*/DH5*α*	2	0.5	>1,024	>1,024	128	32	32	16	4	0.125	0.5	0.5	0.25	64	0.5	16	16/128	4	0.015
pUCP20-*aac3*/DH5*α*	2	4	>1,024	>1,024	128	32	32	16	2	0.125	0.5	0.25	2	8	512	1	128	4	0.015
pUCP20-*aacA4cr*/DH5*α*	2	4	>1,024	>1,024	128	32	32	16	2	0.125	0.5	0.5	4	128	2	8	16	4	<0.0075
pUCP20-*aac(6*′*)-IIc*/DH5*α*	2	4	>1,024	>1,024	128	32	<0.125	16	<1	0.125	0.5	0.015	2	16	0.5	<0.125	4	4	<0.0075
pUCP24-*qnrB4*/DH5*α*	4	4	32	32	2	2	2	1	2	0.125	0.125	0.125	4	4	64	1	1	4	0.015
pUCP24-*qnrB6*/DH5*α*	2	4	16	2	2	2	1	1	<1	0.125	0.125	0.06	4	2	32	1	0.5	32	0.015

FFC: florfenicol; CHL: chloramphenicol; PG: benzylpenicillin; AMP: ampicillin; TIC: ticarcillin; PRL: piperacillin; PTZ: piperacillin/tazobactam; CFZ: cefazolin; FOX: cefoxitin; CAZ: ceftazidime; FEP: cefepime; ATM: aztreonam; STR: streptomycin; KAN: kanamycin; GEN: gentamicin; AMK: amikacin; TOB: tobramycin; NAL: nalidixic acid; CIP: ciprofloxacin.

## Data Availability

The data used to support the findings of this study are included within the article.

## Supplementary Materials

Supplementary Materials Supplementary Table S1: *Citrobacter* isolate information and identification results.
