# Supplementary material for: In Vitro Susceptibility and Florfenicol Resistance in Citrobacter Isolates and Whole-Genome Analysis of Multidrug-Resistant Citrobacter freundii
Source: Int J Genomics. 2019 Nov 19;2019:7191935. doi: 10.1155/2019/7191935 (PMC6885840; doi:10.1155/2019/7191935)
Supplement: Supplementary Materials — Supplementary Table S1: Citrobacter isolate information and identification results. [file 7191935.f1.doc]

**Table S1. *Citrobacter* isolate information and identification results**

| Bacterial code | Source of bacteria | Collection date | Top hit strain (identity with 16S rRNA) |
| --- | --- | --- | --- |
| A14 | Sewage | 2016 | *C. farmeri* CDC 2991-81 (99%) |
| A15 | Sewage | 2016 | *C. farmeri* CDC 2991-81 (99%) |
| F18^a^ | Fish | 2016 | *C. gillenii* CDC 4693-86 (99%) |
| F28 | Fish | 2016 | *C. freundii* ATCC 8090 (99%) |
| F30 | Fish | 2016 | *C. freundii* JCM 1657 (99%) |
| F32 | Fish | 2016 | *C. freundii* JCM 1657 (99%) |
| F39 | Fish | 2016 | *C. freundii* ATCC 8090 (99%) |
| F44^a^ | Fish | 2016 | *C. freundii* JCM 1657 (99%) |
| F47 | Fish | 2016 | *C. freundii* ATCC 8090 (99%) |
| F49 | Fish | 2016 | *C. freundii* ATCC 8090 (99%) |
| F60 | Fish | 2016 | *C. freundii* ATCC 8090 (99%) |
| F61 | Fish | 2016 | *C. freundii* ATCC 8090 (99%) |
| S3 | Soil | 2016 | *C. murliniae* CDC 2970-59 (99%) |
| S16 | Soil | 2016 | *C. murliniae* CDC 2970-59 (99%) |
| S30 | Soil | 2016 | *C. freundii* ATCC 8090 (99%) |
| R44^a^ | Rabbit | 2016 | *C. freundii* JCM 1657 (99%) |
| R47^a^ | Rabbit | 2016 | *C. freundii* JCM 1657 (99%) |
| R49^a^ | Rabbit | 2016 | *C. freundii* JCM 1657 (99%) |
| R51^a^ | Rabbit | 2016 | *C. freundii* JCM 1657 (99%) |
| HXF2^a^ | Fish | 2017 | *C. freundii* ATCC 8090 (99%) |
| HXF4^a^ | Fish | 2017 | *C. murliniae* CDC 2970-59 (99%) |
| HXF6^a^ | Fish | 2017 | *C. freundii* ATCC 8090 (99%) |
| HXF7^a^ | Fish | 2017 | *C. freundii* ATCC 8090 (99%) |
| HXF8^a^ | Fish | 2017 | *C. freundii* ATCC 8090 (99%) |
| HXF10^a^ | Fish | 2017 | *C. murliniae* CDC 2970-59 (99%) |

^a^*floR*-positive isolates.
